# Di-*n*-but­yl{1-[1-(2-hydroxy­phen­yl)ethyl­idene]-5-[1-(2-oxidophen­yl)ethyl­idene]thio­carbazonato-κ^3^ *O* ^5^,*N* ^5^,*S*}tin(IV)

**DOI:** 10.1107/S1600536810016016

**Published:** 2010-05-08

**Authors:** Md. Abu Affan, Dayang N. A. Chee Chee, Zaini Assim, Seik Weng Ng

**Affiliations:** a Faculty of Resource Science and Technology, Universiti Malaysia Sarawak, 94300 Kota Samarahan, Sarawak, Malaysia; b Department of Chemistry, University of Malaya, 50603 Kuala Lumpur, Malaysia

## Abstract

The ‘symmetrical’ 1,5-bis­[1-(2-hydroxy­phen­yl)ethyl­idene]thio­carbazone Schiff base condenses with dibutyl­tin oxide to form the title complex, [Sn(C_4_H_9_)_2_(C_17_H_16_N_4_O_2_S)], in which the deprotonated ligand *O*,*N*,*S*-chelates to the Sn atom of two crystallographically independent mol­ecules. The ligand bears a formal negative charge on the S and one O atom; the other O atom retains its H atom. The Sn atoms are five-coordinated in a *cis*-C_2_NOSSn trigonal-bipyramidal environment, and the apical sites are occupied by the O and S atoms. In both mol­ecules, the hydr­oxy group is hydrogen bonded to a double-bonded N atom, generating a six-membered ring. The amino group is a donor to the coordinated O atom of an adjacent mol­ecule, the hydrogen-bonding inter­action giving rise to a helical chain running along the *b* axis. In one of the independent mol­ecules, the atoms of one of the *n*-butyl groups are disordered over two sets of sites with equal occupancy. In the other independent mol­ecule, the atoms of both *n*-butyl groups are disordered over two sets of sites with equal occupancy and, in addition, the Sn and S atoms were also refined as disordered over two sets of sites with equal occupancy.

## Related literature

For the synthesis of 1,5-bis­(2-hydroxy­benzaldehyde) dithio­carbohydrazone, see: Ren *et al.* (1999 ▶).
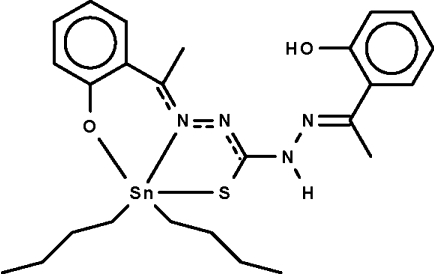


## Experimental

### 

#### Crystal data

[Sn(C_4_H_9_)_2_(C_17_H_16_N_4_O_2_S)]
*M* *_r_* = 573.31
Monoclinic, 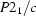

*a* = 24.8858 (17) Å
*b* = 9.4528 (7) Å
*c* = 23.6254 (17) Å
β = 112.756 (1)°
*V* = 5125.0 (6) Å^3^
*Z* = 8
Mo *K*α radiation
μ = 1.11 mm^−1^
*T* = 100 K
0.36 × 0.08 × 0.08 mm

#### Data collection

Bruker SMART APEX diffractometer
Absorption correction: multi-scan *SADABS* (Sheldrick, 1996 ▶) *T*_min_ = 0.691, *T*_max_ = 0.917
47957 measured reflections
11772 independent reflections
7256 reflections with *I* > 2σ(*I*)
*R*_int_ = 0.076

#### Refinement

*R*[*F* ^2^ > 2σ(*F* ^2^)] = 0.058
*wR*(*F* ^2^) = 0.172
*S* = 1.12
11772 reflections
607 parameters
161 restraints
H-atom parameters constrained
Δρ_max_ = 1.77 e Å^−3^
Δρ_min_ = −1.75 e Å^−3^

### 

Data collection: *APEX2* software (Bruker, 2009 ▶); cell refinement: *SAINT* (Bruker, 2009 ▶); data reduction: *SAINT*; program(s) used to solve structure: *SHELXS97* (Sheldrick, 2008 ▶); program(s) used to refine structure: *SHELXL97* (Sheldrick, 2008 ▶); molecular graphics: *X-SEED* (Barbour, 2001 ▶); software used to prepare material for publication: *publCIF* (Westrip, 2010 ▶).

## Supplementary Material

Crystal structure: contains datablocks global, I. DOI: 10.1107/S1600536810016016/lh5030sup1.cif

Structure factors: contains datablocks I. DOI: 10.1107/S1600536810016016/lh5030Isup2.hkl

Additional supplementary materials:  crystallographic information; 3D view; checkCIF report

## Figures and Tables

**Table 1:** Hydrogen-bond geometry (Å, °)

*D*—H⋯*A*	*D*—H	H⋯*A*	*D*⋯*A*	*D*—H⋯*A*
N3—H3⋯O1^i^	0.86	2.24	3.054 (7)	159
N7—H7⋯O3^ii^	0.86	2.21	2.982 (7)	150
O2—H2⋯N4	0.84	1.79	2.503 (8)	141
O4—H4⋯N8	0.84	1.82	2.529 (8)	141

Symmetry codes: (i) 
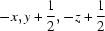
; (ii) 
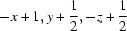
.

**Fig. 1.**
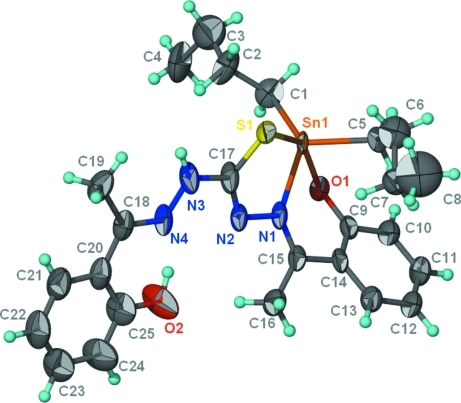
Thermal ellipsoid plot (Barbour, 2001) of one molecule of Sn(C4H9)2(C17H16N4O2S) at the 70% probability level; hydrogen atoms are drawn as spheres of arbitrary radius. The disorder is not shown.

**Fig. 2.**
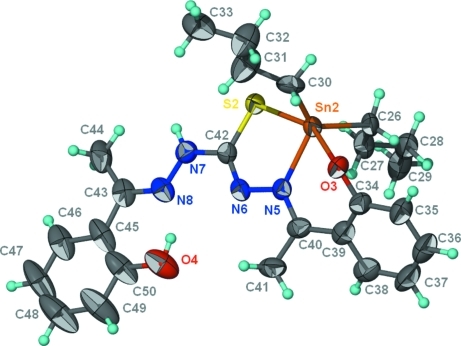
Thermal ellipsoid plot (Barbour, 2001) of second molecule of Sn(C4H9)2(C17H16N4O2S) at the 70% probability level; hydrogen atoms are drawn as spheres of arbitrary radius. The disorder is is not shown.

## supplementary crystallographic information

### Comment

There are many crystallographic studies on metal derivatives of the Schiff bases derived by condensing salicyldehyde (and acetophenone) with semithiocarbazide (and thiocarbazide). 1,5-Bis(2-hydroxyacetophenone) thiocarbazone is a 'symmetrical' ligand; however, in the title di-*n*-butyltin derivative (Scheme I, Fig. 1), the doubly-deprotonated ligand chelates in a *O*,*N*,*S*-manner and only part of the molecule is used in coordination. There are two independent molecules. The tin atoms are five coordinate in a *cis*-C_2_NOSSn trigonal bipyramidal environment, and the apical sites are occupied by the oxgen and sulfur atoms. The hydroxy group is hydrogen bonded to a double-bond nitrogen atom to generate a six-membered ring.

### Experimental

1,5-Bis(2-hydroxyacetophenone) thiocarbazone was synthesized by using a literature method (Ren *et al.*, 1999). The compound (0.66 g, 2 mmole) was dissolved in methanol (20 ml). Potassium hydroxide (0.23 g, 4 mmol) dissolved in methanol (5 ml) was added. The orange solution was then treated with di-*n*-butyltin dichloride (0.61 g, 2 mmol) in methanol (10 ml). The mixture was heated for an hour. The solution was filtered. The evaporation of the solvent gave a product that was recrystallized from ethanol in 60% yield; m.p. 401-403 K.

### Refinement

Hydrogen atoms were placed in calculated positions (C—H 0.93 to 0.97, N–H 0.86, O–H 0.824 Å) and were included in the refinement in the riding model approximation, with *U*(H) set to 1.2 to 1.5*U*_eq_(C,*N*,*O*).

All aromatic rings were refined as rigid hexagons of 1.39 Å sides. Three of the four *n*-butyl chains were refined as two chains of 0.5 occupancy. The 1,2-related distances were restrained to 1.500±0.005 Å and the 1,3-related ones to 2.350±0.005 Å; tight restraints were used, which led to a somewhat large weighting scheme. The temperature factors of the primed atoms were set to those of the unprimed one; the anisotropic temperature factors of the carbon atoms of the chains were restrained to be nearly isotropic.

The Sn1 atom is disordered with respect to Sn1' and the S1 with respect to S1'. Their occupancies were assumed to be 0.5. The anisotropic temperature factors of these four atoms were also restrained to be nearly isotropic. Additionally, the tin–element and tin-element_primed_ distances were restrained to within 0.01 Å of each other. The final difference Fourier map had a peak in the vicinity of the butyl chain belonging to Sn2, and a deep hole in the vicinity of the Sn1/Sn1' atoms.

Some 161 restraints were used to treat the disorder.

### Crystal data

**Table:**

[Sn(C_4_H_9_)_2_(C_17_H_16_N_4_O_2_S)]	*F*(000) = 2352
*M**_r_* = 573.31	*D*_x_ = 1.486 Mg m^−^^3^
Monoclinic, *P*2_1_/*c*	Mo *K*α radiation, λ = 0.71073 Å
Hall symbol: -P 2ybc	Cell parameters from 5611 reflections
*a* = 24.8858 (17) Å	θ = 2.4–24.0°
*b* = 9.4528 (7) Å	µ = 1.11 mm^−^^1^
*c* = 23.6254 (17) Å	*T* = 100 K
β = 112.756 (1)°	Prism, yellow
*V* = 5125.0 (6) Å^3^	0.36 × 0.08 × 0.08 mm
*Z* = 8	

### Data collection

**Table:**

Bruker SMART APEX diffractometer	11772 independent reflections
Radiation source: fine-focus sealed tube	7256 reflections with *I* > 2σ(*I*)
graphite	*R*_int_ = 0.076
ω scans	θ_max_ = 27.5°, θ_min_ = 0.9°
Absorption correction: multi-scan *SADABS* (Sheldrick, 1996)	*h* = −31→32
*T*_min_ = 0.691, *T*_max_ = 0.917	*k* = −12→12
47957 measured reflections	*l* = −30→30

### Refinement

**Table:**

Refinement on *F*^2^	Primary atom site location: structure-invariant direct methods
Least-squares matrix: full	Secondary atom site location: difference Fourier map
*R*[*F*^2^ > 2σ(*F*^2^)] = 0.058	Hydrogen site location: inferred from neighbouring sites
*wR*(*F*^2^) = 0.172	H-atom parameters constrained
*S* = 1.12	*w* = 1/[σ^2^(*F*_o_^2^) + (0.07*P*)^2^ + 7.0*P*] where *P* = (*F*_o_^2^ + 2*F*_c_^2^)/3
11772 reflections	(Δ/σ)_max_ = 0.001
607 parameters	Δρ_max_ = 1.77 e Å^−^^3^
161 restraints	Δρ_min_ = −1.74 e Å^−^^3^

### Fractional atomic coordinates and isotropic or equivalent isotropic displacement parameters (Å^2^)

**Table:**

	*x*	*y*	*z*	*U*_iso_*/*U*_eq_	Occ. (<1)
Sn1	0.08315 (15)	0.8786 (4)	0.24832 (18)	0.0284 (5)	0.50
S1	−0.00074 (19)	1.0465 (5)	0.2565 (2)	0.0426 (12)	0.50
Sn1'	0.08934 (17)	0.8945 (4)	0.26029 (18)	0.0399 (9)	0.50
S1'	−0.00646 (18)	1.0232 (6)	0.22995 (18)	0.0338 (10)	0.50
Sn2	0.415838 (18)	0.26489 (5)	0.24037 (2)	0.03526 (14)	
S2	0.49991 (7)	0.40121 (18)	0.23013 (7)	0.0373 (4)	
O1	0.14093 (17)	0.7062 (4)	0.28324 (19)	0.0347 (10)	
O2	−0.0644 (2)	0.6440 (7)	0.3897 (3)	0.081 (2)	
H2	−0.0642	0.6949	0.3607	0.122*	
O3	0.37888 (18)	0.0927 (5)	0.27034 (19)	0.0386 (10)	
O4	0.6688 (2)	0.0452 (6)	0.4066 (3)	0.0626 (15)	
H4	0.6479	0.0947	0.3767	0.094*	
N1	0.06312 (19)	0.8155 (5)	0.3305 (2)	0.0322 (12)	
N2	0.00592 (19)	0.8274 (5)	0.3265 (2)	0.0329 (12)	
N3	−0.0824 (2)	0.9342 (6)	0.2810 (3)	0.0489 (16)	
H3	−0.1057	0.9948	0.2565	0.059*	
N4	−0.0999 (2)	0.8458 (6)	0.3160 (3)	0.0377 (13)	
N5	0.4907 (2)	0.2071 (5)	0.3249 (2)	0.0307 (11)	
N6	0.5474 (2)	0.2214 (5)	0.3277 (2)	0.0329 (11)	
N7	0.6083 (2)	0.3211 (6)	0.2874 (2)	0.0384 (13)	
H7	0.6151	0.3766	0.2621	0.046*	
N8	0.6519 (2)	0.2451 (6)	0.3305 (2)	0.0389 (13)	
C1	0.0578 (5)	0.7902 (16)	0.1571 (5)	0.040 (2)	0.50
H1A	0.0826	0.8330	0.1375	0.048*	0.50
H1B	0.0662	0.6875	0.1612	0.048*	0.50
C2	−0.0047 (5)	0.8099 (15)	0.1150 (6)	0.058 (3)	0.50
H2A	−0.0303	0.7613	0.1320	0.070*	0.50
H2B	−0.0145	0.9118	0.1111	0.070*	0.50
C3	−0.0139 (7)	0.7497 (15)	0.0534 (5)	0.074 (4)	0.50
H3A	0.0203	0.7684	0.0429	0.088*	0.50
H3B	−0.0488	0.7918	0.0213	0.088*	0.50
C4	−0.0219 (9)	0.5939 (14)	0.0588 (9)	0.074 (4)	0.50
H4A	−0.0315	0.5490	0.0187	0.111*	0.50
H4B	−0.0538	0.5778	0.0728	0.111*	0.50
H4C	0.0141	0.5529	0.0885	0.111*	0.50
C5	0.1541 (7)	1.0249 (16)	0.2874 (9)	0.040 (3)	0.50
H5A	0.1835	0.9767	0.3233	0.048*	0.50
H5B	0.1719	1.0367	0.2568	0.048*	0.50
C6	0.1469 (8)	1.1705 (13)	0.3084 (5)	0.058 (3)	0.50
H6A	0.1786	1.2333	0.3082	0.069*	0.50
H6B	0.1091	1.2115	0.2812	0.069*	0.50
C7	0.1491 (7)	1.1536 (14)	0.3721 (5)	0.043 (2)	0.50
H7A	0.1845	1.1010	0.3981	0.051*	0.50
H7B	0.1143	1.1030	0.3720	0.051*	0.50
C8	0.1507 (13)	1.3038 (19)	0.3944 (10)	0.109 (6)	0.50
H8A	0.1253	1.3633	0.3607	0.164*	0.50
H8B	0.1907	1.3398	0.4089	0.164*	0.50
H8C	0.1370	1.3059	0.4282	0.164*	0.50
C1'	0.0360 (5)	0.8300 (15)	0.1686 (5)	0.040 (2)	0.50
H1'A	0.0080	0.7567	0.1698	0.048*	0.50
H1'B	0.0136	0.9119	0.1451	0.048*	0.50
C2'	0.0737 (4)	0.7725 (17)	0.1381 (5)	0.058 (3)	0.50
H2'A	0.0883	0.6775	0.1545	0.070*	0.50
H2'B	0.1074	0.8356	0.1453	0.070*	0.50
C3'	0.0365 (6)	0.7643 (16)	0.0711 (5)	0.074 (4)	0.50
H3'A	0.0607	0.7421	0.0474	0.088*	0.50
H3'B	0.0165	0.8556	0.0564	0.088*	0.50
C4'	−0.0071 (8)	0.649 (2)	0.0634 (9)	0.074 (4)	0.50
H4'A	0.0129	0.5579	0.0735	0.111*	0.50
H4'B	−0.0354	0.6480	0.0208	0.111*	0.50
H4'C	−0.0275	0.6668	0.0909	0.111*	0.50
C5'	0.1531 (7)	1.0614 (18)	0.2951 (9)	0.040 (3)	0.50
H5'A	0.1928	1.0207	0.3132	0.048*	0.50
H5'B	0.1512	1.1259	0.2614	0.048*	0.50
C6'	0.1398 (6)	1.1410 (16)	0.3431 (7)	0.058 (3)	0.50
H6'A	0.1005	1.1835	0.3251	0.069*	0.50
H6'B	0.1414	1.0769	0.3768	0.069*	0.50
C7'	0.1851 (5)	1.2537 (12)	0.3665 (5)	0.043 (2)	0.50
H7'A	0.1846	1.3147	0.3322	0.051*	0.50
H7'B	0.2242	1.2107	0.3859	0.051*	0.50
C8'	0.1714 (11)	1.339 (2)	0.4125 (11)	0.109 (6)	0.50
H8'A	0.1910	1.4311	0.4181	0.164*	0.50
H8'B	0.1850	1.2889	0.4518	0.164*	0.50
H8'C	0.1292	1.3539	0.3979	0.164*	0.50
C9	0.17920 (14)	0.7085 (4)	0.34316 (13)	0.0325 (14)	
C10	0.23793 (15)	0.6870 (5)	0.35552 (15)	0.0404 (16)	
H10	0.2503	0.6695	0.3228	0.048*	
C11	0.27852 (11)	0.6910 (5)	0.41569 (18)	0.0450 (17)	
H11	0.3187	0.6763	0.4241	0.054*	
C12	0.26040 (14)	0.7167 (5)	0.46350 (13)	0.0414 (16)	
H12	0.2881	0.7195	0.5046	0.050*	
C13	0.20168 (15)	0.7382 (4)	0.45114 (14)	0.0361 (14)	
H13	0.1893	0.7557	0.4838	0.043*	
C14	0.16108 (11)	0.7342 (4)	0.39097 (17)	0.0288 (13)	
C15	0.0988 (2)	0.7536 (6)	0.3813 (3)	0.0304 (13)	
C16	0.0769 (3)	0.7063 (9)	0.4279 (3)	0.0474 (18)	
H16A	0.0428	0.6455	0.4086	0.071*	
H16B	0.0659	0.7889	0.4462	0.071*	
H16C	0.1075	0.6531	0.4600	0.071*	
C17	−0.0251 (3)	0.9210 (7)	0.2872 (3)	0.0446 (17)	
C18	−0.1529 (3)	0.8398 (7)	0.3132 (3)	0.0374 (15)	
C19	−0.2014 (3)	0.9299 (9)	0.2714 (3)	0.0506 (19)	
H19A	−0.1860	0.9958	0.2493	0.076*	
H19B	−0.2187	0.9835	0.2956	0.076*	
H19C	−0.2311	0.8697	0.2419	0.076*	
C20	−0.16271 (19)	0.7323 (5)	0.35400 (19)	0.0415 (16)	
C21	−0.21728 (16)	0.7248 (5)	0.3571 (2)	0.055 (2)	
H21	−0.2480	0.7841	0.3318	0.066*	
C22	−0.22697 (16)	0.6304 (6)	0.3973 (2)	0.058 (2)	
H22	−0.2643	0.6252	0.3994	0.070*	
C23	−0.1821 (2)	0.5435 (5)	0.4344 (2)	0.061 (2)	
H23	−0.1887	0.4790	0.4618	0.073*	
C24	−0.12750 (19)	0.5510 (6)	0.4313 (2)	0.074 (3)	
H24	−0.0968	0.4917	0.4566	0.088*	
C25	−0.11782 (15)	0.6454 (6)	0.3911 (2)	0.054 (2)	
C26	0.3748 (3)	0.4348 (7)	0.2710 (3)	0.0497 (19)	
H26A	0.3517	0.4932	0.2351	0.060*	
H26B	0.3475	0.3928	0.2876	0.060*	
C27	0.4165 (3)	0.5295 (6)	0.3191 (3)	0.0500 (18)	
H27A	0.4430	0.4714	0.3534	0.060*	
H27B	0.4405	0.5829	0.3013	0.060*	
C28	0.3840 (3)	0.6298 (7)	0.3425 (3)	0.056 (2)	
H28A	0.3615	0.5765	0.3620	0.067*	
H28B	0.3562	0.6849	0.3079	0.067*	
C29	0.4256 (3)	0.7286 (7)	0.3884 (4)	0.065 (2)	
H29A	0.4035	0.7980	0.4016	0.097*	
H29B	0.4492	0.7778	0.3696	0.097*	
H29C	0.4511	0.6745	0.4240	0.097*	
C30	0.3902 (8)	0.156 (2)	0.1545 (6)	0.046 (4)	0.50
H30A	0.3766	0.0615	0.1613	0.055*	0.50
H30B	0.3554	0.2064	0.1260	0.055*	0.50
C31	0.4281 (9)	0.131 (4)	0.1196 (12)	0.069 (4)	0.50
H31A	0.4636	0.0782	0.1450	0.082*	0.50
H31B	0.4397	0.2217	0.1069	0.082*	0.50
C32	0.3920 (7)	0.045 (2)	0.0644 (7)	0.062 (4)	0.50
H32A	0.3763	−0.0402	0.0768	0.075*	0.50
H32B	0.3590	0.1021	0.0362	0.075*	0.50
C33	0.4330 (8)	0.006 (3)	0.0343 (9)	0.062 (4)	0.50
H33A	0.4453	0.0913	0.0192	0.093*	0.50
H33B	0.4133	−0.0583	−0.0001	0.093*	0.50
H33C	0.4672	−0.0416	0.0642	0.093*	0.50
C30'	0.3751 (8)	0.190 (2)	0.1485 (5)	0.046 (4)	0.50
H30C	0.3503	0.1079	0.1488	0.055*	0.50
H30D	0.3489	0.2649	0.1237	0.055*	0.50
C31'	0.4132 (7)	0.145 (4)	0.1161 (11)	0.069 (4)	0.50
H31C	0.4400	0.0687	0.1392	0.082*	0.50
H31D	0.4367	0.2259	0.1119	0.082*	0.50
C32'	0.3732 (7)	0.093 (2)	0.0540 (6)	0.062 (4)	0.50
H32C	0.3448	0.0241	0.0576	0.075*	0.50
H32D	0.3518	0.1725	0.0277	0.075*	0.50
C33'	0.4133 (8)	0.024 (3)	0.0284 (9)	0.062 (4)	0.50
H33D	0.4410	0.0939	0.0252	0.093*	0.50
H33E	0.3904	−0.0147	−0.0123	0.093*	0.50
H33F	0.4347	−0.0530	0.0557	0.093*	0.50
C34	0.37953 (19)	0.1006 (5)	0.32815 (15)	0.0406 (16)	
C35	0.32778 (16)	0.0736 (6)	0.33568 (18)	0.0531 (19)	
H35	0.2934	0.0520	0.3010	0.064*	
C36	0.32637 (18)	0.0782 (6)	0.3939 (2)	0.061 (2)	
H36	0.2910	0.0598	0.3990	0.073*	
C37	0.3767 (2)	0.1099 (6)	0.44458 (17)	0.060 (2)	
H37	0.3757	0.1130	0.4844	0.072*	
C38	0.42845 (18)	0.1369 (5)	0.43705 (16)	0.0480 (18)	
H38	0.4629	0.1585	0.4717	0.058*	
C39	0.42986 (15)	0.1322 (5)	0.37884 (19)	0.0393 (15)	
C40	0.4869 (3)	0.1524 (7)	0.3742 (3)	0.0369 (15)	
C41	0.5422 (3)	0.1093 (7)	0.4265 (3)	0.0466 (17)	
H41A	0.5647	0.0459	0.4111	0.070*	
H41B	0.5326	0.0603	0.4578	0.070*	
H41C	0.5655	0.1937	0.4444	0.070*	
C42	0.5535 (3)	0.3044 (7)	0.2865 (3)	0.0337 (14)	
C43	0.7052 (3)	0.2502 (7)	0.3353 (3)	0.0418 (16)	
C44	0.7260 (3)	0.3415 (9)	0.2964 (4)	0.057 (2)	
H44A	0.6928	0.3914	0.2661	0.086*	
H44B	0.7447	0.2825	0.2752	0.086*	
H44C	0.7541	0.4104	0.3224	0.086*	
C45	0.74599 (18)	0.1546 (5)	0.3824 (2)	0.053 (2)	
C46	0.8050 (2)	0.1596 (5)	0.3932 (2)	0.062 (2)	
H46	0.8188	0.2261	0.3718	0.075*	
C47	0.84367 (15)	0.0674 (6)	0.4351 (3)	0.086 (4)	
H47	0.8840	0.0709	0.4424	0.103*	
C48	0.8234 (2)	−0.0299 (5)	0.4664 (2)	0.088 (4)	
H48	0.8499	−0.0929	0.4950	0.106*	
C49	0.7645 (2)	−0.0350 (5)	0.4556 (2)	0.084 (3)	
H49	0.7506	−0.1015	0.4770	0.100*	
C50	0.72574 (16)	0.0572 (5)	0.4137 (2)	0.061 (2)	

### Atomic displacement parameters (Å^2^)

**Table:**

	*U*^11^	*U*^22^	*U*^33^	*U*^12^	*U*^13^	*U*^23^
Sn1	0.0098 (8)	0.0273 (8)	0.0387 (13)	−0.0062 (7)	−0.0008 (10)	0.0071 (8)
S1	0.0235 (17)	0.036 (2)	0.051 (3)	−0.0030 (15)	−0.005 (2)	0.024 (2)
Sn1'	0.0187 (9)	0.0377 (13)	0.0429 (15)	−0.0142 (7)	−0.0107 (8)	0.0185 (11)
S1'	0.0245 (18)	0.048 (2)	0.029 (2)	0.0103 (16)	0.0105 (17)	0.0170 (19)
Sn2	0.0277 (2)	0.0409 (3)	0.0404 (3)	0.00486 (19)	0.01672 (19)	0.0118 (2)
S2	0.0295 (8)	0.0448 (10)	0.0408 (9)	0.0037 (7)	0.0172 (7)	0.0121 (7)
O1	0.021 (2)	0.042 (3)	0.037 (2)	−0.0012 (18)	0.0068 (18)	0.0036 (19)
O2	0.042 (3)	0.093 (5)	0.131 (6)	0.032 (3)	0.057 (4)	0.058 (4)
O3	0.036 (2)	0.045 (3)	0.042 (2)	−0.001 (2)	0.022 (2)	0.008 (2)
O4	0.047 (3)	0.043 (3)	0.073 (4)	−0.008 (2)	−0.005 (3)	0.005 (3)
N1	0.013 (2)	0.025 (3)	0.052 (3)	0.0000 (19)	0.005 (2)	0.003 (2)
N2	0.015 (2)	0.029 (3)	0.050 (3)	0.000 (2)	0.007 (2)	0.003 (2)
N3	0.017 (2)	0.040 (3)	0.082 (4)	0.003 (2)	0.011 (3)	0.018 (3)
N4	0.020 (2)	0.034 (3)	0.057 (3)	−0.003 (2)	0.013 (2)	−0.007 (3)
N5	0.034 (3)	0.024 (3)	0.038 (3)	0.004 (2)	0.019 (2)	0.002 (2)
N6	0.028 (3)	0.033 (3)	0.034 (3)	0.003 (2)	0.009 (2)	−0.004 (2)
N7	0.031 (3)	0.049 (3)	0.035 (3)	−0.005 (3)	0.012 (2)	−0.001 (2)
N8	0.031 (3)	0.038 (3)	0.038 (3)	−0.002 (2)	0.002 (2)	−0.010 (2)
C1	0.039 (6)	0.037 (5)	0.047 (5)	0.014 (4)	0.021 (4)	−0.008 (4)
C2	0.035 (5)	0.064 (6)	0.057 (6)	0.005 (5)	−0.002 (4)	−0.014 (5)
C3	0.058 (6)	0.075 (7)	0.081 (7)	0.006 (6)	0.020 (6)	0.012 (6)
C4	0.049 (7)	0.080 (9)	0.077 (6)	−0.025 (7)	0.008 (6)	−0.024 (7)
C5	0.037 (4)	0.013 (7)	0.056 (5)	−0.009 (4)	0.001 (3)	0.013 (4)
C6	0.061 (6)	0.050 (6)	0.063 (7)	0.004 (5)	0.024 (6)	−0.005 (6)
C7	0.037 (5)	0.040 (5)	0.049 (5)	−0.002 (4)	0.014 (4)	0.007 (4)
C8	0.121 (11)	0.098 (9)	0.118 (10)	0.016 (8)	0.056 (8)	−0.013 (7)
C1'	0.039 (6)	0.037 (5)	0.047 (5)	0.014 (4)	0.021 (4)	−0.008 (4)
C2'	0.035 (5)	0.064 (6)	0.057 (6)	0.005 (5)	−0.002 (4)	−0.014 (5)
C3'	0.058 (6)	0.075 (7)	0.081 (7)	0.006 (6)	0.020 (6)	0.012 (6)
C4'	0.049 (7)	0.080 (9)	0.077 (6)	−0.025 (7)	0.008 (6)	−0.024 (7)
C5'	0.037 (4)	0.013 (7)	0.056 (5)	−0.009 (4)	0.001 (3)	0.013 (4)
C6'	0.061 (6)	0.050 (6)	0.063 (7)	0.004 (5)	0.024 (6)	−0.005 (6)
C7'	0.037 (5)	0.040 (5)	0.049 (5)	−0.002 (4)	0.014 (4)	0.007 (4)
C8'	0.121 (11)	0.098 (9)	0.118 (10)	0.016 (8)	0.056 (8)	−0.013 (7)
C9	0.023 (3)	0.028 (3)	0.042 (3)	−0.005 (2)	0.008 (3)	0.007 (3)
C10	0.027 (3)	0.051 (4)	0.041 (4)	−0.002 (3)	0.011 (3)	0.005 (3)
C11	0.018 (3)	0.060 (5)	0.054 (4)	0.003 (3)	0.011 (3)	0.007 (3)
C12	0.023 (3)	0.054 (4)	0.041 (4)	−0.002 (3)	0.006 (3)	0.005 (3)
C13	0.026 (3)	0.039 (4)	0.042 (3)	0.001 (3)	0.013 (3)	0.001 (3)
C14	0.021 (3)	0.019 (3)	0.043 (3)	−0.006 (2)	0.009 (2)	0.002 (2)
C15	0.022 (3)	0.022 (3)	0.042 (3)	−0.003 (2)	0.006 (2)	−0.002 (3)
C16	0.026 (3)	0.074 (5)	0.040 (4)	−0.003 (3)	0.011 (3)	0.005 (4)
C17	0.017 (3)	0.036 (4)	0.072 (5)	−0.001 (3)	0.008 (3)	0.008 (3)
C18	0.024 (3)	0.045 (4)	0.042 (4)	−0.001 (3)	0.011 (3)	−0.014 (3)
C19	0.024 (3)	0.078 (5)	0.047 (4)	0.010 (3)	0.011 (3)	−0.006 (4)
C20	0.031 (3)	0.051 (4)	0.044 (4)	−0.008 (3)	0.016 (3)	−0.021 (3)
C21	0.031 (4)	0.088 (6)	0.049 (4)	−0.007 (4)	0.018 (3)	−0.009 (4)
C22	0.038 (4)	0.081 (6)	0.066 (5)	−0.011 (4)	0.032 (4)	−0.026 (5)
C23	0.054 (5)	0.082 (6)	0.062 (5)	−0.002 (4)	0.039 (4)	−0.007 (4)
C24	0.051 (5)	0.099 (7)	0.089 (6)	0.019 (5)	0.047 (5)	0.031 (6)
C25	0.040 (4)	0.064 (5)	0.068 (5)	0.012 (4)	0.032 (4)	0.002 (4)
C26	0.043 (4)	0.056 (4)	0.063 (4)	0.025 (3)	0.035 (3)	0.027 (4)
C27	0.045 (4)	0.040 (4)	0.075 (5)	0.009 (3)	0.035 (4)	0.009 (3)
C28	0.059 (4)	0.056 (4)	0.067 (5)	0.016 (4)	0.040 (4)	0.018 (4)
C29	0.079 (5)	0.050 (5)	0.090 (6)	0.010 (4)	0.060 (5)	0.008 (4)
C30	0.038 (8)	0.058 (8)	0.032 (4)	−0.008 (6)	0.001 (4)	0.015 (5)
C31	0.071 (8)	0.074 (7)	0.073 (6)	−0.025 (8)	0.042 (6)	−0.014 (5)
C32	0.065 (8)	0.062 (9)	0.069 (6)	−0.021 (7)	0.037 (6)	−0.019 (6)
C33	0.059 (10)	0.056 (7)	0.051 (5)	−0.003 (8)	0.000 (7)	−0.011 (5)
C30'	0.038 (8)	0.058 (8)	0.032 (4)	−0.008 (6)	0.001 (4)	0.015 (5)
C31'	0.071 (8)	0.074 (7)	0.073 (6)	−0.025 (8)	0.042 (6)	−0.014 (5)
C32'	0.065 (8)	0.062 (9)	0.069 (6)	−0.021 (7)	0.037 (6)	−0.019 (6)
C33'	0.059 (10)	0.056 (7)	0.051 (5)	−0.003 (8)	0.000 (7)	−0.011 (5)
C34	0.046 (4)	0.037 (4)	0.046 (4)	0.005 (3)	0.026 (3)	0.010 (3)
C35	0.052 (4)	0.063 (5)	0.056 (5)	0.001 (4)	0.034 (4)	0.005 (4)
C36	0.062 (5)	0.073 (6)	0.066 (5)	−0.005 (4)	0.044 (5)	0.003 (4)
C37	0.074 (6)	0.068 (6)	0.058 (5)	0.001 (5)	0.047 (5)	0.006 (4)
C38	0.066 (5)	0.034 (4)	0.049 (4)	0.007 (4)	0.028 (4)	0.004 (3)
C39	0.052 (4)	0.026 (3)	0.047 (4)	0.002 (3)	0.028 (3)	0.002 (3)
C40	0.049 (4)	0.028 (3)	0.036 (3)	0.003 (3)	0.019 (3)	0.004 (3)
C41	0.060 (5)	0.042 (4)	0.037 (4)	0.008 (3)	0.017 (3)	0.010 (3)
C42	0.032 (3)	0.037 (4)	0.034 (3)	0.004 (3)	0.015 (3)	−0.001 (3)
C43	0.032 (3)	0.042 (4)	0.045 (4)	−0.009 (3)	0.008 (3)	−0.021 (3)
C44	0.025 (3)	0.080 (6)	0.061 (5)	−0.008 (4)	0.010 (3)	−0.022 (4)
C45	0.032 (4)	0.036 (4)	0.069 (5)	0.003 (3)	−0.005 (3)	−0.028 (4)
C46	0.034 (4)	0.063 (5)	0.070 (5)	−0.002 (4)	−0.002 (4)	−0.034 (4)
C47	0.036 (4)	0.066 (6)	0.110 (8)	0.013 (4)	−0.023 (5)	−0.047 (6)
C48	0.060 (6)	0.047 (5)	0.101 (8)	0.014 (5)	−0.029 (5)	−0.026 (5)
C49	0.053 (5)	0.045 (5)	0.106 (7)	0.002 (4)	−0.021 (5)	−0.007 (5)
C50	0.038 (4)	0.035 (4)	0.077 (6)	−0.003 (3)	−0.014 (4)	−0.016 (4)

### Geometric parameters (Å, °)

**Table:**

Sn1—O1	2.118 (5)	C11—H11	0.9500
Sn1—C5	2.150 (9)	C12—C13	1.3900
Sn1—C1	2.165 (9)	C12—H12	0.9500
Sn1—N1	2.264 (6)	C13—C14	1.3900
Sn1—S1	2.689 (5)	C13—H13	0.9500
S1—C17	1.625 (8)	C14—C15	1.489 (6)
Sn1'—O1	2.138 (5)	C15—C16	1.473 (9)
Sn1'—N1	2.139 (7)	C16—H16A	0.9800
Sn1'—C1'	2.145 (9)	C16—H16B	0.9800
Sn1'—C5'	2.161 (8)	C16—H16C	0.9800
Sn1'—S1'	2.521 (5)	C18—C20	1.484 (8)
S1'—C17	1.862 (8)	C18—C19	1.495 (9)
Sn2—O3	2.122 (4)	C19—H19A	0.9800
Sn2—C30'	2.132 (8)	C19—H19B	0.9800
Sn2—C30	2.140 (8)	C19—H19C	0.9800
Sn2—C26	2.172 (6)	C20—C21	1.3900
Sn2—N5	2.209 (5)	C20—C25	1.3900
Sn2—S2	2.5470 (16)	C21—C22	1.3900
S2—C42	1.736 (6)	C21—H21	0.9500
O1—C9	1.367 (5)	C22—C23	1.3900
O2—C25	1.342 (5)	C22—H22	0.9500
O2—H2	0.8400	C23—C24	1.3900
O3—C34	1.362 (5)	C23—H23	0.9500
O4—C50	1.366 (6)	C24—C25	1.3900
O4—H4	0.8400	C24—H24	0.9500
N1—C15	1.321 (7)	C26—C27	1.502 (5)
N1—N2	1.394 (6)	C26—H26A	0.9900
N2—C17	1.299 (8)	C26—H26B	0.9900
N3—N4	1.358 (8)	C27—C28	1.485 (5)
N3—C17	1.381 (8)	C27—H27A	0.9900
N3—H3	0.8600	C27—H27B	0.9900
N4—C18	1.297 (8)	C28—C29	1.500 (5)
N5—C40	1.313 (8)	C28—H28A	0.9900
N5—N6	1.393 (7)	C28—H28B	0.9900
N6—C42	1.303 (8)	C29—H29A	0.9800
N7—C42	1.366 (8)	C29—H29B	0.9800
N7—N8	1.370 (7)	C29—H29C	0.9800
N7—H7	0.8600	C30—C31	1.492 (5)
N8—C43	1.288 (8)	C30—H30A	0.9900
C1—C2	1.497 (5)	C30—H30B	0.9900
C1—H1A	0.9900	C31—C32	1.500 (5)
C1—H1B	0.9900	C31—H31A	0.9900
C2—C3	1.494 (5)	C31—H31B	0.9900
C2—H2A	0.9900	C32—C33	1.500 (5)
C2—H2B	0.9900	C32—H32A	0.9900
C3—C4	1.499 (5)	C32—H32B	0.9900
C3—H3A	0.9900	C33—H33A	0.9800
C3—H3B	0.9900	C33—H33B	0.9800
C4—H4A	0.9800	C33—H33C	0.9800
C4—H4B	0.9800	C30'—C31'	1.491 (5)
C4—H4C	0.9800	C30'—H30C	0.9900
C5—C6	1.497 (5)	C30'—H30D	0.9900
C5—H5A	0.9900	C31'—C32'	1.502 (5)
C5—H5B	0.9900	C31'—H31C	0.9900
C6—C7	1.492 (5)	C31'—H31D	0.9900
C6—H6A	0.9900	C32'—C33'	1.501 (5)
C6—H6B	0.9900	C32'—H32C	0.9900
C7—C8	1.511 (5)	C32'—H32D	0.9900
C7—H7A	0.9900	C33'—H33D	0.9800
C7—H7B	0.9900	C33'—H33E	0.9800
C8—H8A	0.9800	C33'—H33F	0.9800
C8—H8B	0.9800	C34—C35	1.3900
C8—H8C	0.9800	C34—C39	1.3900
C1'—C2'	1.488 (5)	C35—C36	1.3900
C1'—H1'A	0.9900	C35—H35	0.9500
C1'—H1'B	0.9900	C36—C37	1.3900
C2'—C3'	1.495 (5)	C36—H36	0.9500
C2'—H2'A	0.9900	C37—C38	1.3900
C2'—H2'B	0.9900	C37—H37	0.9500
C3'—C4'	1.497 (5)	C38—C39	1.3900
C3'—H3'A	0.9900	C38—H38	0.9500
C3'—H3'B	0.9900	C39—C40	1.477 (7)
C4'—H4'A	0.9800	C40—C41	1.507 (9)
C4'—H4'B	0.9800	C41—H41A	0.9800
C4'—H4'C	0.9800	C41—H41B	0.9800
C5'—C6'	1.499 (5)	C41—H41C	0.9800
C5'—H5'A	0.9900	C43—C45	1.488 (8)
C5'—H5'B	0.9900	C43—C44	1.489 (10)
C6'—C7'	1.492 (5)	C44—H44A	0.9800
C6'—H6'A	0.9900	C44—H44B	0.9800
C6'—H6'B	0.9900	C44—H44C	0.9800
C7'—C8'	1.498 (5)	C45—C46	1.3900
C7'—H7'A	0.9900	C45—C50	1.3900
C7'—H7'B	0.9900	C46—C47	1.3900
C8'—H8'A	0.9800	C46—H46	0.9500
C8'—H8'B	0.9800	C47—C48	1.3900
C8'—H8'C	0.9800	C47—H47	0.9500
C9—C10	1.3900	C48—C49	1.3900
C9—C14	1.3900	C48—H48	0.9500
C10—C11	1.3900	C49—C50	1.3900
C10—H10	0.9500	C49—H49	0.9500
C11—C12	1.3900		
			
O1—Sn1—C5	90.4 (6)	C13—C14—C15	117.1 (3)
O1—Sn1—C1	89.1 (4)	C9—C14—C15	122.9 (3)
C5—Sn1—C1	123.4 (6)	N1—C15—C16	120.1 (5)
O1—Sn1—N1	78.7 (2)	N1—C15—C14	119.2 (5)
C5—Sn1—N1	102.6 (5)	C16—C15—C14	120.8 (5)
C1—Sn1—N1	132.5 (5)	C15—C16—H16A	109.5
O1—Sn1—S1	148.8 (2)	C15—C16—H16B	109.5
C5—Sn1—S1	96.9 (5)	H16A—C16—H16B	109.5
C1—Sn1—S1	111.3 (4)	C15—C16—H16C	109.5
N1—Sn1—S1	70.05 (18)	H16A—C16—H16C	109.5
C17—S1—Sn1	92.0 (3)	H16B—C16—H16C	109.5
O1—Sn1'—N1	81.1 (2)	N2—C17—N3	116.2 (6)
O1—Sn1'—C1'	95.5 (4)	N2—C17—S1	126.5 (5)
N1—Sn1'—C1'	115.4 (4)	N3—C17—S1	115.8 (5)
O1—Sn1'—C5'	103.4 (6)	N2—C17—S1'	128.8 (5)
N1—Sn1'—C5'	111.0 (5)	N3—C17—S1'	114.4 (5)
C1'—Sn1'—C5'	131.9 (7)	N4—C18—C20	114.7 (6)
O1—Sn1'—S1'	152.0 (3)	N4—C18—C19	123.6 (6)
N1—Sn1'—S1'	81.4 (2)	C20—C18—C19	121.7 (5)
C1'—Sn1'—S1'	72.7 (3)	C18—C19—H19A	109.5
C5'—Sn1'—S1'	103.3 (6)	C18—C19—H19B	109.5
C17—S1'—Sn1'	91.0 (3)	H19A—C19—H19B	109.5
O3—Sn2—C30'	89.3 (7)	C18—C19—H19C	109.5
O3—Sn2—C30	86.5 (7)	H19A—C19—H19C	109.5
C30'—Sn2—C30	12.6 (9)	H19B—C19—H19C	109.5
O3—Sn2—C26	97.8 (2)	C21—C20—C25	120.0
C30'—Sn2—C26	119.2 (5)	C21—C20—C18	118.6 (4)
C30—Sn2—C26	131.7 (5)	C25—C20—C18	121.3 (4)
O3—Sn2—N5	80.31 (17)	C22—C21—C20	120.0
C30'—Sn2—N5	137.7 (5)	C22—C21—H21	120.0
C30—Sn2—N5	125.1 (5)	C20—C21—H21	120.0
C26—Sn2—N5	102.9 (2)	C21—C22—C23	120.0
O3—Sn2—S2	153.89 (12)	C21—C22—H22	120.0
C30'—Sn2—S2	99.0 (7)	C23—C22—H22	120.0
C30—Sn2—S2	96.2 (6)	C24—C23—C22	120.0
C26—Sn2—S2	99.59 (18)	C24—C23—H23	120.0
N5—Sn2—S2	76.94 (13)	C22—C23—H23	120.0
C42—S2—Sn2	94.3 (2)	C23—C24—C25	120.0
C9—O1—Sn1	119.1 (3)	C23—C24—H24	120.0
C9—O1—Sn1'	111.0 (3)	C25—C24—H24	120.0
C25—O2—H2	109.5	O2—C25—C24	116.4 (4)
C34—O3—Sn2	117.0 (3)	O2—C25—C20	123.6 (4)
C50—O4—H4	109.5	C24—C25—C20	120.0
C15—N1—N2	114.0 (5)	C27—C26—Sn2	114.6 (4)
C15—N1—Sn1'	124.1 (4)	C27—C26—H26A	108.6
N2—N1—Sn1'	121.9 (4)	Sn2—C26—H26A	108.6
C15—N1—Sn1	127.1 (4)	C27—C26—H26B	108.6
N2—N1—Sn1	118.6 (4)	Sn2—C26—H26B	108.6
C17—N2—N1	114.4 (5)	H26A—C26—H26B	107.6
N4—N3—C17	116.1 (5)	C28—C27—C26	110.2 (5)
N4—N3—H3	122.0	C28—C27—H27A	109.6
C17—N3—H3	122.0	C26—C27—H27A	109.6
C18—N4—N3	123.7 (6)	C28—C27—H27B	109.6
C40—N5—N6	114.6 (5)	C26—C27—H27B	109.6
C40—N5—Sn2	125.1 (4)	H27A—C27—H27B	108.1
N6—N5—Sn2	120.3 (4)	C27—C28—C29	110.1 (5)
C42—N6—N5	115.4 (5)	C27—C28—H28A	109.6
C42—N7—N8	116.8 (5)	C29—C28—H28A	109.6
C42—N7—H7	121.6	C27—C28—H28B	109.6
N8—N7—H7	121.6	C29—C28—H28B	109.6
C43—N8—N7	122.6 (6)	H28A—C28—H28B	108.1
C2—C1—Sn1	116.2 (8)	C28—C29—H29A	109.5
C2—C1—H1A	108.2	C28—C29—H29B	109.5
Sn1—C1—H1A	108.2	H29A—C29—H29B	109.5
C2—C1—H1B	108.2	C28—C29—H29C	109.5
Sn1—C1—H1B	108.2	H29A—C29—H29C	109.5
H1A—C1—H1B	107.4	H29B—C29—H29C	109.5
C3—C2—C1	109.2 (7)	C31—C30—Sn2	125.5 (11)
C3—C2—H2A	109.8	C31—C30—H30A	105.9
C1—C2—H2A	109.8	Sn2—C30—H30A	105.9
C3—C2—H2B	109.8	C31—C30—H30B	105.9
C1—C2—H2B	109.8	Sn2—C30—H30B	105.9
H2A—C2—H2B	108.3	H30A—C30—H30B	106.3
C2—C3—C4	105.4 (7)	C30—C31—C32	105.8 (7)
C2—C3—H3A	110.7	C30—C31—H31A	110.6
C4—C3—H3A	110.7	C32—C31—H31A	110.6
C2—C3—H3B	110.7	C30—C31—H31B	110.6
C4—C3—H3B	110.7	C32—C31—H31B	110.6
H3A—C3—H3B	108.8	H31A—C31—H31B	108.7
C3—C4—H4A	109.5	C33—C32—C31	104.9 (7)
C3—C4—H4B	109.5	C33—C32—H32A	110.8
H4A—C4—H4B	109.5	C31—C32—H32A	110.8
C3—C4—H4C	109.5	C33—C32—H32B	110.8
H4A—C4—H4C	109.5	C31—C32—H32B	110.8
H4B—C4—H4C	109.5	H32A—C32—H32B	108.8
C6—C5—Sn1	123.4 (11)	C32—C33—H33A	109.5
C6—C5—H5A	106.5	C32—C33—H33B	109.5
Sn1—C5—H5A	106.5	H33A—C33—H33B	109.5
C6—C5—H5B	106.5	C32—C33—H33C	109.5
Sn1—C5—H5B	106.5	H33A—C33—H33C	109.5
H5A—C5—H5B	106.5	H33B—C33—H33C	109.5
C7—C6—C5	105.8 (7)	C31'—C30'—Sn2	118.1 (10)
C7—C6—H6A	110.6	C31'—C30'—H30C	107.8
C5—C6—H6A	110.6	Sn2—C30'—H30C	107.8
C7—C6—H6B	110.6	C31'—C30'—H30D	107.8
C5—C6—H6B	110.6	Sn2—C30'—H30D	107.8
H6A—C6—H6B	108.7	H30C—C30'—H30D	107.1
C6—C7—C8	103.8 (7)	C30'—C31'—C32'	106.3 (7)
C6—C7—H7A	111.0	C30'—C31'—H31C	110.5
C8—C7—H7A	111.0	C32'—C31'—H31C	110.5
C6—C7—H7B	111.0	C30'—C31'—H31D	110.5
C8—C7—H7B	111.0	C32'—C31'—H31D	110.5
H7A—C7—H7B	109.0	H31C—C31'—H31D	108.7
C7—C8—H8A	109.5	C33'—C32'—C31'	104.1 (7)
C7—C8—H8B	109.5	C33'—C32'—H32C	110.9
H8A—C8—H8B	109.5	C31'—C32'—H32C	110.9
C7—C8—H8C	109.5	C33'—C32'—H32D	110.9
H8A—C8—H8C	109.5	C31'—C32'—H32D	110.9
H8B—C8—H8C	109.5	H32C—C32'—H32D	109.0
C2'—C1'—Sn1'	109.5 (7)	C32'—C33'—H33D	109.5
C2'—C1'—H1'A	109.8	C32'—C33'—H33E	109.5
Sn1'—C1'—H1'A	109.8	H33D—C33'—H33E	109.5
C2'—C1'—H1'B	109.8	C32'—C33'—H33F	109.5
Sn1'—C1'—H1'B	109.8	H33D—C33'—H33F	109.5
H1'A—C1'—H1'B	108.2	H33E—C33'—H33F	109.5
C1'—C2'—C3'	106.5 (7)	O3—C34—C35	117.8 (3)
C1'—C2'—H2'A	110.4	O3—C34—C39	122.2 (3)
C3'—C2'—H2'A	110.4	C35—C34—C39	120.0
C1'—C2'—H2'B	110.4	C36—C35—C34	120.0
C3'—C2'—H2'B	110.4	C36—C35—H35	120.0
H2'A—C2'—H2'B	108.6	C34—C35—H35	120.0
C2'—C3'—C4'	106.8 (7)	C35—C36—C37	120.0
C2'—C3'—H3'A	110.4	C35—C36—H36	120.0
C4'—C3'—H3'A	110.4	C37—C36—H36	120.0
C2'—C3'—H3'B	110.4	C38—C37—C36	120.0
C4'—C3'—H3'B	110.4	C38—C37—H37	120.0
H3'A—C3'—H3'B	108.6	C36—C37—H37	120.0
C3'—C4'—H4'A	109.5	C37—C38—C39	120.0
C3'—C4'—H4'B	109.5	C37—C38—H38	120.0
H4'A—C4'—H4'B	109.5	C39—C38—H38	120.0
C3'—C4'—H4'C	109.5	C38—C39—C34	120.0
H4'A—C4'—H4'C	109.5	C38—C39—C40	117.5 (4)
H4'B—C4'—H4'C	109.5	C34—C39—C40	122.4 (4)
C6'—C5'—Sn1'	108.1 (7)	N5—C40—C39	121.2 (5)
C6'—C5'—H5'A	110.1	N5—C40—C41	118.5 (6)
Sn1'—C5'—H5'A	110.1	C39—C40—C41	120.3 (5)
C6'—C5'—H5'B	110.1	C40—C41—H41A	109.5
Sn1'—C5'—H5'B	110.1	C40—C41—H41B	109.5
H5'A—C5'—H5'B	108.4	H41A—C41—H41B	109.5
C7'—C6'—C5'	106.5 (6)	C40—C41—H41C	109.5
C7'—C6'—H6'A	110.4	H41A—C41—H41C	109.5
C5'—C6'—H6'A	110.4	H41B—C41—H41C	109.5
C7'—C6'—H6'B	110.4	N6—C42—N7	117.5 (6)
C5'—C6'—H6'B	110.4	N6—C42—S2	127.9 (5)
H6'A—C6'—H6'B	108.6	N7—C42—S2	114.6 (5)
C6'—C7'—C8'	107.7 (7)	N8—C43—C45	115.0 (6)
C6'—C7'—H7'A	110.2	N8—C43—C44	124.0 (7)
C8'—C7'—H7'A	110.2	C45—C43—C44	121.0 (6)
C6'—C7'—H7'B	110.2	C43—C44—H44A	109.5
C8'—C7'—H7'B	110.2	C43—C44—H44B	109.5
H7'A—C7'—H7'B	108.5	H44A—C44—H44B	109.5
C7'—C8'—H8'A	109.5	C43—C44—H44C	109.5
C7'—C8'—H8'B	109.5	H44A—C44—H44C	109.5
H8'A—C8'—H8'B	109.5	H44B—C44—H44C	109.5
C7'—C8'—H8'C	109.5	C46—C45—C50	120.0
H8'A—C8'—H8'C	109.5	C46—C45—C43	118.8 (4)
H8'B—C8'—H8'C	109.5	C50—C45—C43	121.2 (4)
O1—C9—C10	118.0 (3)	C45—C46—C47	120.0
O1—C9—C14	122.0 (3)	C45—C46—H46	120.0
C10—C9—C14	120.0	C47—C46—H46	120.0
C11—C10—C9	120.0	C48—C47—C46	120.0
C11—C10—H10	120.0	C48—C47—H47	120.0
C9—C10—H10	120.0	C46—C47—H47	120.0
C10—C11—C12	120.0	C49—C48—C47	120.0
C10—C11—H11	120.0	C49—C48—H48	120.0
C12—C11—H11	120.0	C47—C48—H48	120.0
C11—C12—C13	120.0	C50—C49—C48	120.0
C11—C12—H12	120.0	C50—C49—H49	120.0
C13—C12—H12	120.0	C48—C49—H49	120.0
C12—C13—C14	120.0	O4—C50—C49	115.8 (4)
C12—C13—H13	120.0	O4—C50—C45	124.2 (4)
C14—C13—H13	120.0	C49—C50—C45	120.0
C13—C14—C9	120.0		
			
O1—Sn1—S1—C17	−29.4 (6)	O1—C9—C14—C13	−178.8 (4)
C5—Sn1—S1—C17	−131.7 (7)	C10—C9—C14—C13	0.0
C1—Sn1—S1—C17	98.2 (6)	O1—C9—C14—C15	3.2 (5)
N1—Sn1—S1—C17	−30.8 (3)	C10—C9—C14—C15	−178.0 (4)
O1—Sn1'—S1'—C17	44.1 (6)	N2—N1—C15—C16	−0.9 (8)
N1—Sn1'—S1'—C17	−7.9 (3)	Sn1'—N1—C15—C16	178.2 (5)
C1'—Sn1'—S1'—C17	112.2 (5)	Sn1—N1—C15—C16	−173.7 (5)
C5'—Sn1'—S1'—C17	−117.6 (6)	N2—N1—C15—C14	179.8 (4)
O3—Sn2—S2—C42	−14.4 (4)	Sn1'—N1—C15—C14	−1.1 (7)
C30'—Sn2—S2—C42	−121.5 (6)	Sn1—N1—C15—C14	7.0 (8)
C30—Sn2—S2—C42	−109.1 (6)	C13—C14—C15—N1	148.4 (4)
C26—Sn2—S2—C42	116.7 (3)	C9—C14—C15—N1	−33.6 (7)
N5—Sn2—S2—C42	15.6 (2)	C13—C14—C15—C16	−30.9 (7)
C5—Sn1—O1—C9	47.8 (6)	C9—C14—C15—C16	147.2 (5)
C1—Sn1—O1—C9	171.3 (5)	N1—N2—C17—N3	178.9 (6)
N1—Sn1—O1—C9	−54.9 (4)	N1—N2—C17—S1	−16.0 (9)
S1—Sn1—O1—C9	−56.3 (6)	N1—N2—C17—S1'	8.2 (9)
C5—Sn1—O1—Sn1'	41.1 (15)	N4—N3—C17—N2	−1.2 (9)
C1—Sn1—O1—Sn1'	164.5 (16)	N4—N3—C17—S1	−168.0 (5)
N1—Sn1—O1—Sn1'	−61.6 (14)	N4—N3—C17—S1'	170.8 (5)
S1—Sn1—O1—Sn1'	−63.0 (13)	Sn1—S1—C17—N2	37.7 (7)
N1—Sn1'—O1—C9	−61.3 (3)	Sn1—S1—C17—N3	−157.1 (6)
C1'—Sn1'—O1—C9	−176.2 (5)	Sn1—S1—C17—S1'	−66.5 (7)
C5'—Sn1'—O1—C9	48.3 (6)	Sn1'—S1'—C17—N2	2.8 (7)
S1'—Sn1'—O1—C9	−113.3 (5)	Sn1'—S1'—C17—N3	−168.0 (5)
N1—Sn1'—O1—Sn1	112.4 (15)	Sn1'—S1'—C17—S1	93.5 (7)
C1'—Sn1'—O1—Sn1	−2.5 (14)	N3—N4—C18—C20	178.1 (5)
C5'—Sn1'—O1—Sn1	−138.0 (16)	N3—N4—C18—C19	−0.2 (10)
S1'—Sn1'—O1—Sn1	60.4 (14)	N4—C18—C20—C21	175.8 (4)
C30'—Sn2—O3—C34	−165.5 (6)	C19—C18—C20—C21	−5.8 (7)
C30—Sn2—O3—C34	−177.8 (6)	N4—C18—C20—C25	−0.7 (7)
C26—Sn2—O3—C34	−46.1 (4)	C19—C18—C20—C25	177.6 (5)
N5—Sn2—O3—C34	55.7 (4)	C25—C20—C21—C22	0.0
S2—Sn2—O3—C34	85.3 (4)	C18—C20—C21—C22	−176.6 (5)
O1—Sn1'—N1—C15	38.7 (5)	C20—C21—C22—C23	0.0
C1'—Sn1'—N1—C15	130.6 (6)	C21—C22—C23—C24	0.0
C5'—Sn1'—N1—C15	−62.3 (8)	C22—C23—C24—C25	0.0
S1'—Sn1'—N1—C15	−163.3 (5)	C23—C24—C25—O2	−178.5 (6)
O1—Sn1'—N1—N2	−142.2 (4)	C23—C24—C25—C20	0.0
C1'—Sn1'—N1—N2	−50.4 (6)	C21—C20—C25—O2	178.4 (7)
C5'—Sn1'—N1—N2	116.8 (7)	C18—C20—C25—O2	−5.1 (7)
S1'—Sn1'—N1—N2	15.8 (4)	C21—C20—C25—C24	0.0
O1—Sn1'—N1—Sn1	−78.0 (13)	C18—C20—C25—C24	176.5 (5)
C1'—Sn1'—N1—Sn1	13.9 (12)	O3—Sn2—C26—C27	117.1 (5)
S1'—Sn1'—N1—Sn1	80.0 (13)	C30'—Sn2—C26—C27	−149.4 (9)
O1—Sn1—N1—C15	27.7 (5)	C30—Sn2—C26—C27	−150.7 (10)
C5—Sn1—N1—C15	−60.1 (7)	N5—Sn2—C26—C27	35.3 (5)
C1—Sn1—N1—C15	105.9 (7)	S2—Sn2—C26—C27	−43.4 (5)
S1—Sn1—N1—C15	−153.0 (5)	Sn2—C26—C27—C28	−172.8 (5)
O1—Sn1—N1—N2	−144.8 (4)	C26—C27—C28—C29	−177.5 (6)
C5—Sn1—N1—N2	127.4 (6)	O3—Sn2—C30—C31	−132 (3)
C1—Sn1—N1—N2	−66.6 (6)	C30'—Sn2—C30—C31	125 (7)
S1—Sn1—N1—N2	34.5 (4)	C26—Sn2—C30—C31	130 (2)
O1—Sn1—N1—Sn1'	95.7 (13)	N5—Sn2—C30—C31	−57 (3)
C5—Sn1—N1—Sn1'	7.9 (13)	S2—Sn2—C30—C31	22 (3)
C1—Sn1—N1—Sn1'	173.9 (16)	Sn2—C30—C31—C32	177.5 (18)
S1—Sn1—N1—Sn1'	−85.0 (13)	C30—C31—C32—C33	−173 (2)
C15—N1—N2—C17	161.2 (6)	O3—Sn2—C30'—C31'	−117 (2)
Sn1'—N1—N2—C17	−17.9 (7)	C30—Sn2—C30'—C31'	−40 (5)
Sn1—N1—N2—C17	−25.4 (7)	C26—Sn2—C30'—C31'	144 (2)
C17—N3—N4—C18	−176.3 (6)	N5—Sn2—C30'—C31'	−43 (3)
O3—Sn2—N5—C40	−31.0 (5)	S2—Sn2—C30'—C31'	38 (2)
C30'—Sn2—N5—C40	−109.0 (11)	Sn2—C30'—C31'—C32'	178.1 (19)
C30—Sn2—N5—C40	−109.7 (9)	C30'—C31'—C32'—C33'	−169 (2)
C26—Sn2—N5—C40	64.9 (5)	Sn2—O3—C34—C35	130.8 (3)
S2—Sn2—N5—C40	161.9 (5)	Sn2—O3—C34—C39	−50.2 (5)
O3—Sn2—N5—N6	146.0 (4)	O3—C34—C35—C36	179.0 (4)
C30'—Sn2—N5—N6	67.9 (11)	C39—C34—C35—C36	0.0
C30—Sn2—N5—N6	67.3 (9)	C34—C35—C36—C37	0.0
C26—Sn2—N5—N6	−118.2 (4)	C35—C36—C37—C38	0.0
S2—Sn2—N5—N6	−21.1 (4)	C36—C37—C38—C39	0.0
C40—N5—N6—C42	−164.4 (5)	C37—C38—C39—C34	0.0
Sn2—N5—N6—C42	18.4 (7)	C37—C38—C39—C40	−176.0 (5)
C42—N7—N8—C43	178.9 (6)	O3—C34—C39—C38	−179.0 (5)
O1—Sn1—C1—C2	145.0 (12)	C35—C34—C39—C38	0.0
C5—Sn1—C1—C2	−125.1 (13)	O3—C34—C39—C40	−3.2 (5)
N1—Sn1—C1—C2	71.3 (13)	C35—C34—C39—C40	175.8 (5)
S1—Sn1—C1—C2	−10.7 (13)	N6—N5—C40—C39	179.1 (5)
Sn1—C1—C2—C3	177.0 (9)	Sn2—N5—C40—C39	−3.8 (8)
C1—C2—C3—C4	82.0 (15)	N6—N5—C40—C41	−1.4 (8)
O1—Sn1—C5—C6	−150.2 (13)	Sn2—N5—C40—C41	175.7 (4)
C1—Sn1—C5—C6	120.6 (11)	C38—C39—C40—N5	−151.4 (5)
N1—Sn1—C5—C6	−71.7 (14)	C34—C39—C40—N5	32.7 (7)
S1—Sn1—C5—C6	−0.7 (13)	C38—C39—C40—C41	29.1 (7)
Sn1—C5—C6—C7	84.2 (16)	C34—C39—C40—C41	−146.9 (5)
C5—C6—C7—C8	171.9 (16)	N5—N6—C42—N7	−179.7 (5)
O1—Sn1'—C1'—C2'	−46.9 (11)	N5—N6—C42—S2	1.5 (8)
N1—Sn1'—C1'—C2'	−129.6 (10)	N8—N7—C42—N6	2.0 (8)
C5'—Sn1'—C1'—C2'	66.6 (14)	N8—N7—C42—S2	−179.0 (4)
S1'—Sn1'—C1'—C2'	159.1 (11)	Sn2—S2—C42—N6	−15.6 (6)
Sn1'—C1'—C2'—C3'	−166.0 (9)	Sn2—S2—C42—N7	165.6 (4)
C1'—C2'—C3'—C4'	−69.5 (18)	N7—N8—C43—C45	−176.8 (5)
O1—Sn1'—C5'—C6'	−118.4 (14)	N7—N8—C43—C44	2.0 (10)
N1—Sn1'—C5'—C6'	−32.9 (17)	N8—C43—C45—C46	−176.2 (4)
C1'—Sn1'—C5'—C6'	131.3 (13)	C44—C43—C45—C46	5.0 (7)
S1'—Sn1'—C5'—C6'	52.8 (16)	N8—C43—C45—C50	6.6 (7)
Sn1'—C5'—C6'—C7'	179.3 (12)	C44—C43—C45—C50	−172.3 (5)
C5'—C6'—C7'—C8'	177.5 (17)	C50—C45—C46—C47	0.0
Sn1—O1—C9—C10	−127.2 (3)	C43—C45—C46—C47	−177.3 (5)
Sn1'—O1—C9—C10	−126.2 (3)	C45—C46—C47—C48	0.0
Sn1—O1—C9—C14	51.6 (4)	C46—C47—C48—C49	0.0
Sn1'—O1—C9—C14	52.6 (4)	C47—C48—C49—C50	0.0
O1—C9—C10—C11	178.8 (4)	C48—C49—C50—O4	−179.5 (5)
C14—C9—C10—C11	0.0	C48—C49—C50—C45	0.0
C9—C10—C11—C12	0.0	C46—C45—C50—O4	179.5 (6)
C10—C11—C12—C13	0.0	C43—C45—C50—O4	−3.3 (6)
C11—C12—C13—C14	0.0	C46—C45—C50—C49	0.0
C12—C13—C14—C9	0.0	C43—C45—C50—C49	177.2 (5)
C12—C13—C14—C15	178.1 (4)		

### Hydrogen-bond geometry (Å, °)

**Table:**

*D*—H···*A*	*D*—H	H···*A*	*D*···*A*	*D*—H···*A*
N3—H3···O1^i^	0.86	2.24	3.054 (7)	159
N7—H7···O3^ii^	0.86	2.21	2.982 (7)	150
O2—H2···N4	0.84	1.79	2.503 (8)	141
O4—H4···N8	0.84	1.82	2.529 (8)	141

Symmetry codes: (i) −*x*, *y*+1/2, −*z*+1/2; (ii) −*x*+1, *y*+1/2, −*z*+1/2.
